# Elevated brain glutamine levels in adults with autism spectrum disorder: A 7T MRS study

**DOI:** 10.1038/s41380-025-03440-z

**Published:** 2025-12-27

**Authors:** Manabu Kubota, Yujiro Yoshihara, Teruhisa Uwatoko, Rin Shoji, Jamie Near, Masoumeh Dehghani, Yuta Y. Aoki, Shin-ichi Urayama, Tomohisa Okada, Toshiya Murai

**Affiliations:** 1 Department of Psychiatry, Graduate School of Medicine, Kyoto University, Kyoto, Japan; 2 University Health Center, Kyoto University of Education, Kyoto, Japan; 3 Physical Sciences, Sunnybrook Research Institute, Toronto, Ontario Canada; 4 Department of Medical Biophysics, University of Toronto, Toronto, Ontario Canada; 5 Department of Psychiatry, Aoki Clinic, Tokyo, Japan; 6 Human Brain Research Center, Graduate School of Medicine, Kyoto University, Kyoto, Japan

**Keywords:** Autism spectrum disorders, Neuroscience

## Abstract

Alterations in excitatory neurotransmitters, involving the glutamate (Glu) and glutamine (Gln) cycle, as well as inhibitory neurotransmission, GABA, are implicated in the pathophysiology of autism spectrum disorder (ASD). Although magnetic resonance spectroscopy (MRS) holds promise for assessing these metabolites, conventional 3 T MRI does not robustly measure them, leaving the neurochemical pathophysiology of ASD insufficiently understood. 7 T MRI enables reliable assessments of these neurometabolites by enhancing the signal-to-noise ratio and improving the spectral resolution, particularly in distinguishing neuroactive Glu from its metabolic precursor, Gln. The current 7 T MRS study has two primary objectives: first, to investigate neurometabolite levels in adults with ASD to elucidate its neurochemical pathophysiology, and second, to examine their association with symptoms of ASD. Thirty-three adults with ASD (mean age = 31 years) and 52 age-matched control adults were included. The neurometabolite levels of Glu, Gln, and GABA were assessed in the anterior cingulate cortex (ACC), thalamus, and right temporo-parietal junction (TPJ), with most quantifications passing quality checks. Analysis of covariance revealed significant effects of diagnosis on Gln in the thalamus (*p* = 0.008) and right TPJ (*p* = 0.006), indicating elevated Gln levels in these regions in the ASD group. Among social communication and restricted and repetitive behaviors, significant negative correlations were observed in the ASD group between Gln levels and sensory symptoms. These findings suggest that alterations in the excitatory neurotransmission regulation, presumably increased cycling of the Gln-Glu circuit, may underlie the pathophysiology of ASD.

## Introduction

Autism spectrum disorder (ASD) is a neurodevelopmental condition characterized by difficulties in social communication and interaction, as well as restricted and repetitive patterns of behaviors, interests, or activities (RRB). In addition to these core symptoms, individuals with ASD frequently exhibit specific cognitive characteristics, including weak central coherence, hyper-systemization, a stick-to-the-sameness, and atypical sensory processing [1]. However, despite these diverse symptoms, their underlying neurobiological mechanisms remain unclear.

Among the various hypotheses that have been proposed for the pathophysiology of ASD, alterations in excitatory and inhibitory neurotransmission states have been prominently implicated [2, 3]. Within the central nervous system, glutamate (Glu) functions as the primary excitatory neurotransmitter released from presynaptic terminals. To rapidly clear Glu from the synapse and prevent excitotoxicity, it is taken up by astrocytes and participates in a recycling cycle with its metabolic precursor, glutamine (Gln). Gln is synthesized and stored by astrocytes and is subsequently transferred back to neurons, serving as the precursor for replenishing the neuronal Glu supply [4, 5] (further details in Discussion). In contrast, inhibitory neurotransmission is mediated by γ-aminobutyric acid (GABA). Prior genetic and postmortem studies have revealed abnormalities in these neurotransmission systems in ASD: genetic studies have revealed altered single-nucleotide polymorphisms (SNPs) in genes related to Glu transporters [6], and postmortem studies have demonstrated the overexpression of mRNA encoding Glu receptors [7]. In addition, altered plasma Gln levels [8–10] have been reported in ASD compared to controls. Regarding inhibitory neurotransmission, researchers have identified atypical SNPs in genes associated with glutamate decarboxylase (GAD) and atypical copy number variations affecting GABA_A_ receptor-associated genetic sites [11, 12]. Consistent with the findings of genetic studies, postmortem surveys have demonstrated lower expression of mRNA associated with GAD and GABA_A_ receptors in individuals with ASD compared to controls [13]. Beyond these findings regarding Glu and GABA, cumulative evidence has indicated dysfunction of the Gln-Glu cycle and the excitatory versus inhibitory balance (E/I balance). For instance, genetic studies have identified atypical SNPs regulating the Gln-Glu cycle [14], and postmortem analyses have shown a lower expression of enzymes involved in the cycle [15]. Additionally, electroencephalography studies involving individuals with ASD have demonstrated atypical gamma oscillations, and magnetic resonance imaging (MRI) studies have revealed atypical local connectivity, both indicative of a dysregulated E/I balance [3, 16, 17]. Furthermore, a prior preclinical investigation revealed that the Gln-Glu cycle is pathologically upregulated in a murine model of autism, contributing to E/I imbalance and the manifestation of autism-like behaviors [18].

Despite such indirect evidence suggesting the involvement of atypical Glu, Gln, and GABA levels, in vivo quantification of these metabolites in individuals with ASD has encountered substantial challenges. Magnetic resonance spectroscopy (MRS) facilitates non-invasive measurement of these metabolites; however, at magnetic field strengths up to 3 T, Glu and Gln are frequently measured as a combined signal referred to as “Glx” due to their low spectral resolution [19, 20]. Notably, previous MRS studies in ASD, predominantly conducted using 1.5–3 T MR scanners, have not succeeded in quantifying the metabolite levels, resulting in inconsistent findings [21–26]. Our literature review [25–27] in combination with updated search identified approximately 80 published MRS studies on ASD. Of these, 63% used 3 T and 35% employed 1.5 T MRI, respectively (as of October 2025; for our search strategy, see Supplementary Methods S1 and Supplementary Results S1). Among studies employing higher magnetic field strengths, only one research group has published data focusing on children with ASD using 4 T MRI, reporting solely on Glu [28, 29]. To date, no published 7 T MRS studies were identified in the context of ASD. This limitation poses a significant challenge to investigations of the neural mechanisms underlying ASD, given the distinct roles of Glu and Gln in the Gln-Glu cycle within the central nervous system. Indeed, a recent meta-analysis of MRS studies reported no significant differences in the levels of Glx (*n* = 17), Glu (*n* = 4), or GABA (*n* = 11) in adults with ASD, with Gln excluded due to insufficient study numbers [25]. Utilizing MRS with ultra-high-field MRI, such as 7 T MRI, addresses this limitation by enhancing the spectral resolution, thereby allowing differentiation of Glu and Gln and even GABA signals [19, 20, 30].

The present 7 T MRS study has two primary objectives: (1) to compare brain metabolite levels in ASD with controls, and (2) to investigate the association between neurotransmission disturbances and ASD symptomatology. We selected the anterior cingulate cortex (ACC), thalamus, and right temporoparietal junction (rTPJ) for the MRS experiment, considering the roles of these regions in social function and sensory processing [31–33]. Based on the aforementioned literature on ASD pathophysiology, we hypothesized that adults with ASD would show evidence of a dysregulated Gln-Glu cycle and heightened E/I balance, manifested by an increase in Gln levels, accompanied by potential alterations in Glu and lower GABA levels. We further predicted that the identified neurometabolite alterations would be associated with the severity of ASD symptomatology.

## Materials and methods

### Participants

A total of 33 adults with ASD and 52 neurotypical control adults were included in this study. The sample size was determined based on prior MRS studies conducted on individuals with ASD [25–27]. The participants with ASD were recruited from the Department of Psychiatry at Kyoto University Hospital and affiliated clinics. The diagnosis of ASD was made according to the criteria outlined in the fifth edition text revision of the Diagnostic and Statistical Manual of Mental Disorders (DSM-5). The evaluation involved interviewing participants concerning their developmental history, present illness, life history, and family history. They were also requested to be accompanied by a family member or another individual who had known them since early childhood. The diagnosis of ASD was confirmed through a consensus reached by board-certified psychiatrists, including at least one psychiatrist with specialization in neurodevelopmental disorders, in conjunction with a clinical psychologist. Twenty-two participants in the ASD group were taking psychotropic medication (some taking more than one class): antidepressants (*n* = 10), benzodiazepines and/or hypnotics (*n* = 13), antipsychotics (*n* = 6), mood stabilizers (*n* = 5), and ADHD medication (*n* = 6). Neurotypical control participants were recruited from the local community through advertisements and acquaintances. The control participants did not meet criteria for any psychiatric disorders according to the Structured Clinical Interview for DSM-5 (SCID) conducted by a board-certified psychiatrist. None of the control participants had a history of serious medical or surgical illnesses, neurodevelopmental disorders, or substance abuse.

This study was approved by the Committee on Medical Ethics of Kyoto University and was conducted in accordance with the Code of Ethics of the World Medical Association. After providing a complete description of the study, written informed consent was obtained from all participants.

### Clinical measures

The intelligence quotient (IQ) scores of the individuals with ASD had been assessed using either Wechsler Adult Intelligence Scale-Third Edition (WAIS-III), WAIS-Fourth Edition (WAIS-IV), or WAIS-Revised (WAIS-R). An exception was noted for one participant with ASD, who was in her early 20 s at the time of participation. Her score, obtained at age 15, was evaluated using the Wechsler Intelligence Scale for Children-Fourth Edition (WISC-IV), which is validated against WAIS-IV [34]. The IQ scores of the control participants were estimated using the Japanese version of the National Adult Reading Test (JART) based on previous findings that JART successfully predicted full-scale IQ scores in controls [35, 36]. Autistic traits were assessed with Autism Spectrum Quotient (AQ) [37, 38].

The Social Responsiveness Scale, second edition (SRS-2) adult self-report form was used to assess the severity of ASD-related symptoms [39]. Higher scores on SRS-2 indicate greater levels of autistic traits.

The Adolescent/Adult Sensory Profile (AASP) was used to assess sensory characteristics [40]. AASP is a self-rating questionnaire that assesses the sensory categories of taste/smell, movement, vision, touch, activity level, hearing, exploration, sensory sensitivity, and sensation avoidance. It includes four subscales: Low Registration, Sensation Seeking, Sensory Sensitivity, and Sensation Avoiding. Higher scores in these subscales indicate a stronger tendency toward atypical sensory processing.

### Image acquisition

Structural T1-weighted images of all subjects were acquired with a 7-T MRI scanner (MAGNETOM 7 T, Siemens Healthcare, Erlangen, Germany. System VB17A) equipped with a whole-body gradient system (80 mT/m maximum amplitude, 200 mT/m/ms maximum slew rate). A single-channel transmit and 32-channel receiver head coil (Nova Medical, MA, USA) was used. The images were acquired using magnetization-prepared 2 rapid acquisition gradient echo (MP2RAGE, research prototype sequence) [41] with the following parameters: TR, 6000 ms; TE, 2.9 ms; TI, 800 and 2700 ms; flip angle, 4 and 5 degrees; number of slices, 256; slice thickness, 0.7 mm; field of view, 195 × 198 mm; matrix size, 280 × 284; spatial resolution, 0.7 × 0.7 × 0.7 mm.

MRS scans were conducted using the semi-adiabatic short TE spin-echo full-intensity-acquired localized single voxel spectroscopy (sSPECIAL) sequence [42, 43]. The volumes of interest (VOIs) were localized at the ACC (30 × 20 × 20 mm^3^), the thalamus (30 × 20 × 15 mm^3^), and the rTPJ (30 × 20 × 30 mm^3^). For the rTPJ, the VOI were centered and aligned with the Sylvian fissure. This positioning encompassed the superior temporal gyrus, supramarginal gyrus, angular gyrus, and posterior superior temporal sulcus, while ensuring that the VOI remained posterior to the postcentral gyrus, consistent with previous literature [44, 45]. The parameters for the MRS scans are: TE: 16 ms, TR: 7000 (ACC, thalamus) or 8000 (rTPJ) ms. 64 averages were collected using VAPOR water suppression, and 4 additional averages were acquired with the water suppression RF pulses turned off. After performing automatic shimming using the Fast Automatic Shim Technique with Echo-planar Signal readout for Mapping Along Projections (FASTEST MAP, Siemens prototype sequence), manual shimming was performed to make the linewidth of the water spectrum smaller than 20 Hz. All MRS scans were conducted between morning and noon to minimize potential effects of circadian rhythms on neurometabolite levels [46, 47]. In addition, participants were strongly encouraged to fast on the day of the MR scan to minimize potential effects of food intake [48, 49] (one individual with ASD could not follow the instruction and had a light meal).

### MRS data processing

MRS data were processed in MATLAB (Natick MA, USA) using the FID-A toolkit [50]. First, a weighted combination of receiver channels was conducted, followed by removal of motion-corrupted signal averages, frequency and phase drift correction using spectral registration [51], and subspectra alignment before subtraction and signal averaging were performed.

MRS data analysis was carried out with LCModel (ver. 6.3-1 N, Stephen Provencher Inc., Oakville, Ontario, Canada), using a “basis-set” of simulated model spectra for the specific 7 T sSPECIAL sequence. This study assessed the Glu, Gln, and GABA levels. Tissue composition inside the VOIs was calculated based on the T1-weighted image segmentation using Gannet 3.0. Water concentrations were calculated based on the volume fractions of white matter (WM), gray matter (GM), and cerebrospinal fluid (CSF), assuming their water concentrations as 35,880 mM, 43,300 mM, and 55,556 mM, respectively [52]. Metabolite concentrations were then divided by the WM and GM fractions to correct for CSF inside the VOI [53].

The signal-to-noise ratio (SNR) was obtained using a N-acetylaspartate (NAA) peak height of 2.01 ppm divided by the standard deviation (S.D.) of noise. MRS Spectra quality was ensured by including only data with SNR > 10, full-width at half maximum (FWHM) < 0.05 ppm, and Cramér–Rao lower bound (CRLB) ≦ 20%.

**Fig. 1:**
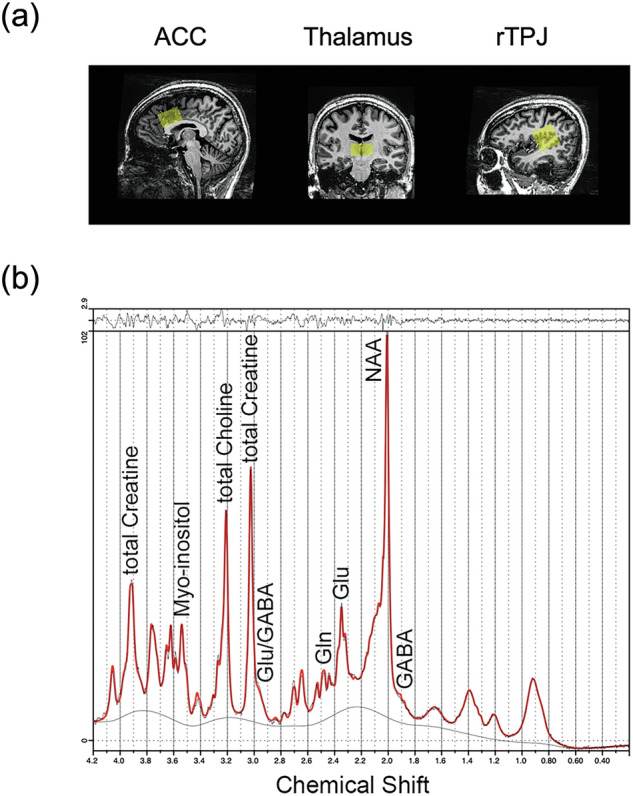
Representative VOI images and a representative spectral fit for MRS analysis. (**a**) Representative images of ACC, thalamus, and rTPJ VOIs used for the MRS experiment. (**b**) A representative spectral fitting of MRS spectrum to LCModel. ACC anterior cingulate cortex, GABA γ-aminobutyric acid, Gln glutamine, Glu glutamate, NAA N-acetylaspartate, rTPJ right temporo-parietal junction, MRS magnetic resonance spectroscopy, VOI volume of interest.

Representative images of VOIs and a representative LCModel spectral fit are shown in Fig. 1.

### Statistical analysis

All statistical analyses were conducted with SPSS 27.0 (SPSS Inc.).

#### Comparisons in behavioral, clinical measures and in neurometabolite levels between ASD and control groups

First, independent sample t-tests were conducted to examine differences in behavioral measures, total scores of AQ and SRS-2, and the four subscale scores of AASP between the ASD and control groups. Statistical significance thresholds were set at *p* < 0.05.

Subsequently, analysis of covariance (ANCOVA) was conducted to examine the effects of diagnosis on Glu, Gln, and GABA levels in each VOI, with age and sex included as covariates. Statistical significance thresholds were set at *p* < 0.05 with Bonferroni correction for the three VOIs.

#### Correlational analysis between neurometabolite levels and clinical measures

Initially, we conducted separate correlational analyses within the ASD and control groups to examine the relationships between neurometabolite levels (Glu, Gln, and GABA) and the total SRS-2 score, as well as the four subscale scores of the AASP. Subsequently, as our main purpose, we investigated whether the association between the neurometabolite levels and clinical measures differed significantly between the groups.

Pearson’s r was employed for correlational analyses in each group. However, for analyses involving non-normally distributed ACC GABA levels in controls, Spearman’s rho was employed. Statistical significance thresholds were set at *p* < 0.05 with Bonferroni correction for the three VOIs.

In cases where significant correlations were identified, two-tailed t-tests were employed to compare z-transformed correlation coefficients (using Fisher’s transformation) of the neurometabolite levels and the clinical scale scores between the two diagnostic groups, with a statistical significance of *p* < 0.05.

#### Analysis of ratios of Gln-to-Glu (Gln/Glu) and Glu-to-GABA (Glu/GABA)

As measures of Gln-Glu cycling and E/I balance, respectively [54–56], mean Gln/Glu and Glu/GABA ratios were calculated across the three VOIs (ACC, thalamus, and rTPJ) for each subject. ANCOVA, controlling for age and sex, was employed to examine diagnostic effects on these ratios. Subsequently, their correlations with the SRS-2 total and AASP subscale scores were assessed. In cases of significant correlations, further investigations were conducted by comparing z-transformed correlation coefficients between the two diagnostic groups. Because of the exploratory nature of these analyses, statistical significance levels were set at *p* < 0.05.

## Results

### Behavioral and clinical measures

The demographic information of the participants is presented in Table 1. There were no significant differences in age, sex, education level, or estimated IQ between the ASD and control groups. Comorbidity was observed in 16 individuals with ASD (ten with ADHD, three with depression, one with anxiety disorder, one with schizophrenia, and one with mild intellectual disability), while none had comorbid bipolar disorder or substance use disorder according to SCID. Total AQ and SRS-2 scores were significantly higher in the ASD group. The ASD group exhibited significantly higher scores on three of the four subscales of AASP (Low Registration, Sensory Sensitivity, and Sensation Avoiding), and significantly lower scores on the remaining subscale (Sensation Seeking).

**Table 1:** Characteristics of individuals included in this study.

	ASD (*N* = 33)		Controls (*N* = 52)		Statistics	
	Mean	S.D.	Mean	S.D.	t/chi-square	p
Age (years)	31.1	9.7	35.6	12.2	1.89	0.06
Sex (male/female)	18/15		32/20		0.41	0.65
Education (years)	15.0	2.4	15.3	2.2	0.70	0.49
Estimated IQ	105.7	20.5	109.0	6.6	0.90	0.37
AQ	30.6	6.5	18.0	8.3	−7.39	<0.001^1)^
SRS-2	103.0	25.7	59.3	28.7	−7.13	<0.001^1)^
AASP						
Low Registration	39.8	7.5	28.2	8.9	−6.26	<0.001^1)^
Sensation Seeking	34.8	5.9	39.1	8.7	2.68	0.009^1)^
Sensory Sensitivity	39.8	9.2	33.9	8.2	−3.10	0.003^1)^
Sensation Avoiding	40.8	8.3	34.2	9.0	−3.39	0.001^1)^

^1)^*p* < 0.05.

*AASP* adolescent/adult sensory profile, *AQ* autism spectrum quotient, *ASD* autism spectrum disorder, *IQ* intelligence quotient, *S.D*. standard deviation, *SRS-2* social responsiveness scale, second edition adult self-report form.

### MRS quantification

The proportions of neurometabolite data meeting our quality control criteria were as follows: Glu levels, ACC 98.8%, Thalamus 87.1%, rTPJ 94.1%; Gln levels, ACC 97.6%, Thalamus 82.4%, rTPJ 92.9%; GABA levels, ACC 91.8%, Thalamus 72.4%, rTPJ 83.5%. No significant differences were observed between the ASD and control groups in these metrics. Supplementary Table S1 shows the SNR and CRLB for Glu, Gln, and GABA in each VOI following the MRS quality controls. The average SNR values in each VOI were substantially higher than those reported in the majority of MRS studies [23, 57].

### Group comparisons in Glu, Gln, and GABA levels

**Fig. 2:**
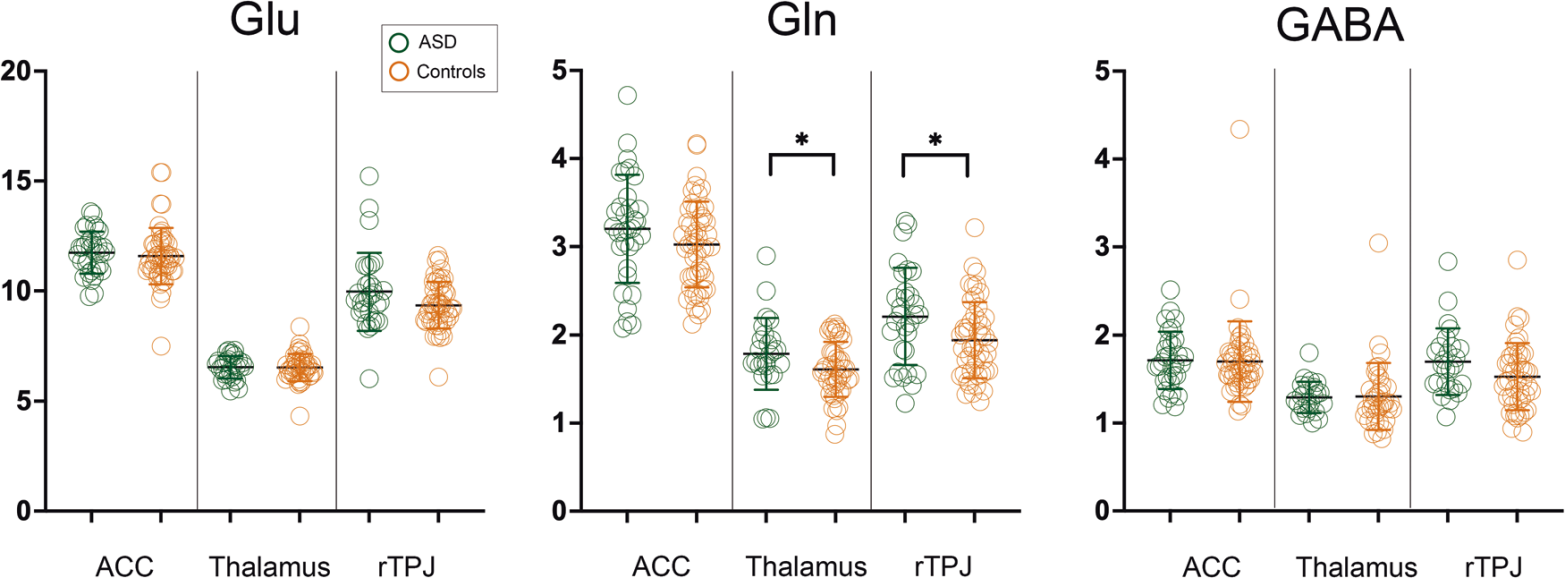
Group comparisons of Glu, Gln, and GABA levels between the ASD and control groups. Measured values are presented in institutional units. Error bars represent standard deviation. *p < 0.05/3. ACC anterior cingulate cortex, ASD autism spectrum disorder, Gln glutamine, Glu glutamate, GABA γ-aminobutyric acid, rTPJ right temporo-parietal junction.

ANCOVA revealed significant diagnostic effects on Gln levels in the thalamus (*p* = 0.008) and the rTPJ (*p* = 0.006), indicating higher Gln levels in these regions in individuals with ASD (Table 2, Fig. 2). Our post-hoc analyses revealed no significant effects of psychotropic medication use or the presence of comorbid ADHD or depression on Gln levels in these regions (Supplementary Methods S2, S3 and Supplementary Results S2, S3). On the other hand, no significant diagnostic effects were observed for Glu or GABA levels. The exclusion of an outlier (one control subject) in the analysis of ACC GABA levels, which exceeded three times the interquartile range of the boxplot (also see Fig. 2), did not materially change the results.

**Table 2:** Comparisons in neurometabolite levels between the ASD and control groups.

	ASD (N = 33)			Controls (N = 52)			Effect of diagnosis	
	n	Mean	S.D.	n	Mean	S.D.	F	p
Glu levels								
ACC	33	11.74	0.96	51	11.59	1.27	0.35	0.55
Thalamus	29	6.55	0.51	45	6.50	0.55	0.00	0.95
rTPJ	30	9.97	1.76	50	9.32	1.05	2.59	0.11
Gln levels								
ACC	33	3.20	0.61	50	3.03	0.49	4.32	0.04
Thalamus	26	1.79	0.41	44	1.60	0.31	7.51	0.008^1)^
rTPJ	30	2.21	0.55	49	1.94	0.44	7.97	0.006^1)^
GABA levels								
ACC	31	1.71	0.33	47	1.70	0.46	0.00	0.98
Thalamus	24	1.29	0.18	38	1.29	0.37	0.06	0.80
rTPJ	26	1.70	0.38	45	1.52	0.38	2.39	0.13

Mean and S.D. values are presented in institutional units.

*ACC* anterior cingulate cortex, *ASD* autism spectrum disorder, *GABA* γ-aminobutyric acid, *Gln* glutamine, *Glu* glutamate, *rTPJ* right temporo-parietal junction, *S.D*. standard deviation.

^1)^*p* < 0.05/3.

These results on the thalamic and rTPJ Gln levels remained significant after excluding one individual with comorbid schizophrenia from the ASD group and another with comorbid mild intellectual disability.

Excluding one participant with ASD who did not fast on the day of the MRS scan did not materially alter the results (please note that neurometabolite measures in the thalamus of this participant were already excluded by our quality check).

### Correlational analysis between neurometabolite levels and clinical measures

**Fig. 3:**
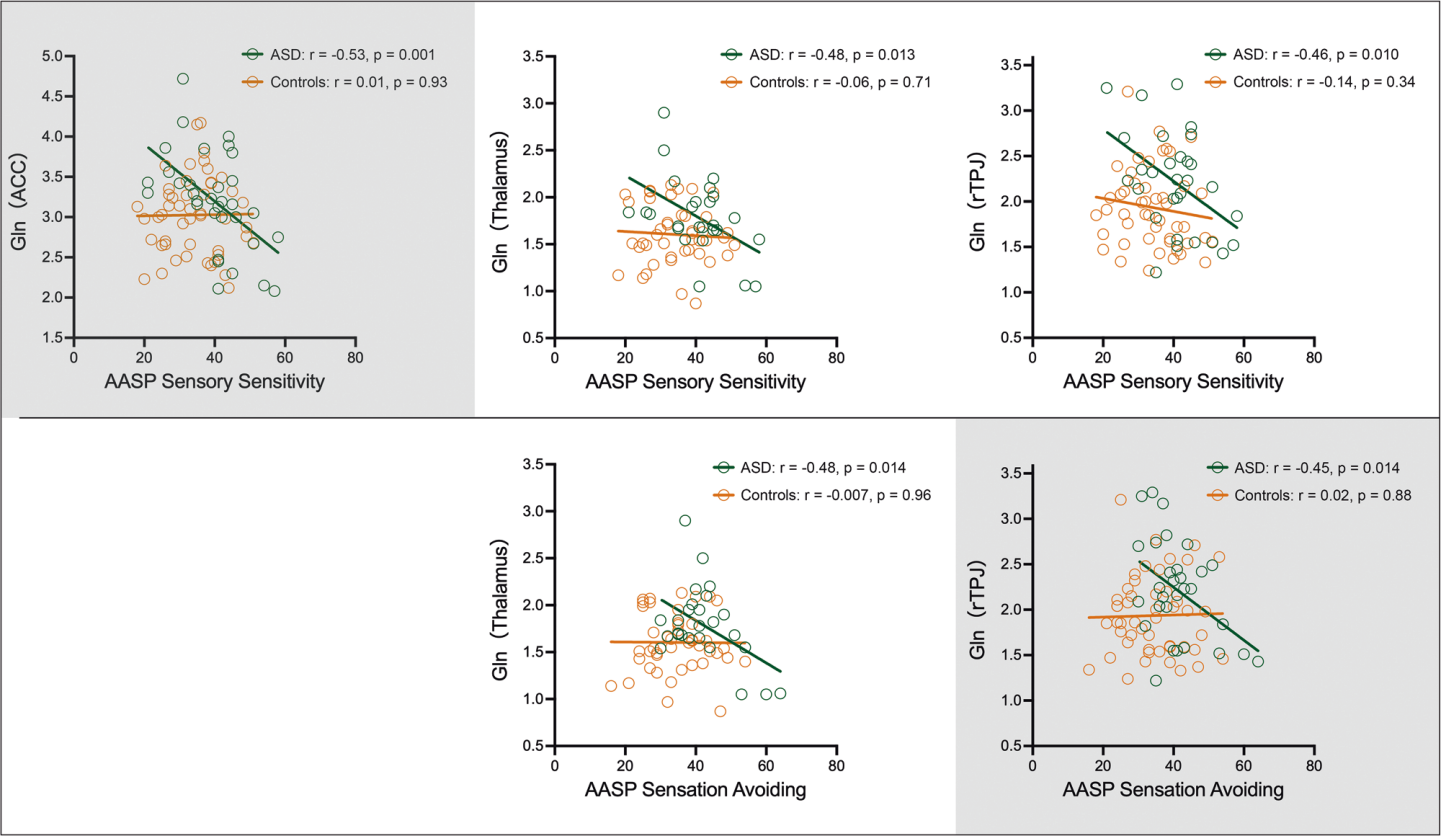
Scatter plots of Gln levels against AASP subscale scores for the ASD and control groups. Measured metabolite values are presented in institutional units. Plots with a gray background indicate those in which the correlation coefficients differed significantly between the ASD and control groups, based on Fisher’s z-transformation. Significant group differences were observed in the relationships between 1) ACC Gln levels and AASP Sensory Sensitivity (z = 2.6, *p* = 0.009), and 2) rTPJ Gln levels and AASP Sensation Avoiding (z = 2.1, *p* = 0.04). AASP Adolescent/Adult Sensory Profile, ACC anterior cingulate cortex, ASD autism spectrum disorder, Gln glutamine, r Pearson’s r, rTPJ right temporo-parietal junction.

Gln levels in several regions were negatively correlated with the AASP Sensory Sensitivity and Sensation Avoiding subscale scores only in the ASD group (for details, see Fig. 3 and Supplementary Results S1). These correlations remained significant after controlling for medication use (Supplementary Methods S2 and Supplementary Results S2).

Comparisons of z-transformed correlation coefficients between the ASD and control groups revealed significant group differences in the relationships between 1) ACC Gln levels and AASP Sensory Sensitivity (z = 2.6, *p* = 0.009) and 2) rTPJ Gln levels and AASP Sensation Avoiding (z = 2.1, *p* = 0.04), indicating that these associations differed significantly between the ASD and control groups (Fig. 3). This pattern remained unchanged even after excluding one participant with comorbid schizophrenia and another with comorbid mild intellectual disability.

### Analysis of mean Gln/Glu and Glu/GABA ratios

ANCOVA demonstrated a significant diagnostic effect on the Gln/Glu ratio, indicating a higher Gln/Glu ratio in the ASD group (*p* = 0.016; Supplementary Table S2, Supplementary Figure S1), while no significant diagnostic effect was found for the Glu/GABA ratio. Our post-hoc analyses revealed no significant effects of medication use or the presence of comorbid ADHD or depression on the Gln/Glu ratio (Supplementary Methods S2, S3 and Supplementary Results S2, S3).

Significant negative correlations were observed between the Gln/Glu ratio and AASP Sensory Sensitivity (r = −0.59, *p* = 0.002) and Sensation Avoiding (r = −0.47, *p* = 0.019) subscale scores within the ASD group (Supplementary Fig. S2). These correlations remained significant after controlling for medication intake (Supplementary Methods S2 and Supplementary Results S2). No other significant correlations were identified in either group, for either Gln/Glu or Glu/GABA ratios.

Comparisons of z-transformed correlation coefficients revealed a significant group difference (ASD vs. controls) in the association between the Gln/Glu ratio and AASP Sensory Sensitivity scores (z = 2.3, *p* = 0.02) (Supplementary Fig. S2).

## Discussion

In this 7 T MRS study, significantly higher Gln levels were revealed in the thalamus and rTPJ of adults with ASD, compared to controls. Significant group differences were observed in the relationship between ACC Gln levels and AASP Sensory Sensitivity scores, and between rTPJ Gln levels and AASP Sensation Avoiding scores, in which negative correlations were found for the ASD group only. Furthermore, the ASD group exhibited a significantly higher Gln/Glu ratio compared to controls, which was negatively correlated with both Sensory Sensitivity and Sensation Avoiding subscales.

Multiple lines of evidence have suggested the involvement of dysregulation in the excitatory and inhibitory neurotransmission in the pathophysiology of ASD. Nonetheless, MRS studies investigating neurometabolite levels have yielded inconsistent results [25]. Our investigations, benefiting from a larger sample size and improved SNRs due to a stronger magnetic field, revealed elevated levels of Gln (a precursor of Glu) and the Gln/Glu ratio in adults with ASD. On the other hand, no significant differences were found in the Glu or GABA levels, or in the Glu/GABA ratio between the ASD and control groups, which is in line with a recent meta-analysis of MRS studies in adults with ASD [25].

In neural mechanisms, Glu is released into the synapse, where it facilitates excitatory neurotransmission in the postsynaptic neuron. Subsequently, Glu is absorbed by astrocytes, where it is converted into Gln by the enzyme glutamine synthetase (GS). While astrocytes primarily synthesize Gln, neurons uptake Gln, which is then reconverted into Glu by glutaminase [4]. Given that Glu is primarily neuroactive while Gln is storable and neuroinactive, the Gln/Glu ratio can reflect the efficiency of Glu uptake by astrocytes and its conversion to Gln, which is crucial for regulating excitatory neurotransmission [20]. A fraction of Gln in astrocytes is transported into GABAergic neurons, where it is transformed into Glu (alternatively, Gln in astrocytes is converted into Glu and then transported into GABAergic neurons), which is ultimately converted into GABA by glutamate decarboxylase [4]. Binding of GABA to GABA_A_ receptor induces the opening of the ligand-gated chloride channel, leading to an increased influx of chloride ions, which results in hyperpolarization. Although both Glu and GABA exist in stored and released forms, the Glu/GABA ratio is widely used as an indicator of the overall E/I balance [56].

The elevated Gln levels and Gln/Glu ratio observed in our adult ASD group may reflect an altered Gln-Glu cycle, potentially driven by modifications in astrocyte function or Gln transport, as suggested by previous postmortem studies [15]. This could suggest an overactive Gln-Glu cycle in adults with ASD, which may represent either a manifestation of, or a compensatory response to, dysregulated excitatory neurotransmission implicated in the pathophysiology of ASD. Given the significant elevation in the thalamus and rTPJ, this altered cycle may be particularly pronounced in these regions compared to the ACC. Contrary to our hypothesis, we found no significant differences in Glu or GABA levels, nor in the Glu/GABA ratio, between the ASD and control groups. This absence of significant differences suggests that primary abnormalities in the direct metabolic pathways involving Glu and GABA were not evident in our adult sample. Notably, our results on GABA align with several prior studies using optimized sequences such as MEGA-PRESS [25], suggesting that methodological sensitivity is unlikely to be the sole factor responsible for the non-significant findings. However, we cannot exclude the possibility that GABA pathology might exist outside the VOIs we assessed. Additionally, it is possible that dynamic dysfunction of the inhibitory system might be present despite the static nature of GABA levels as measured by MRS [58, 59].

Our cross-sectional design limits our ability to determine whether these observed changes represent primary pathophysiology originating in childhood ASD or compensatory mechanisms developing during adulthood. However, previous research in animal models of ASD has shown increased glutamate receptor binding during adolescence instead of childhood, potentially reflecting compensatory mechanisms for earlier synaptic alterations [60]. This suggests that our observed elevation in Gln levels and Gln/Glu ratio in adults with ASD might, at least in part, represent a compensatory adaptation within the glutamatergic system aimed at modulating its function. Therefore, future longitudinal studies integrating clinical and preclinical approaches are warranted to elucidate the mechanistic interplay among various components of excitatory glutamatergic transmission across the lifespan of individuals with ASD.

Sensory symptoms were found to be negatively correlated with the Gln levels and the Gln/Glu ratio only in the ASD group, and specifically with the Sensory Sensitivity and Sensation Avoiding subscale scores of AASP. Comparative analysis of the correlation coefficients revealed that negative correlations between ACC Gln levels and Sensory Sensitivity, between rTPJ Gln levels and Sensation Avoiding, and between the Gln/Glu ratio and Sensory Sensitivity were unique to the ASD group: lower values in these neurometabolite measures were associated with increases in the severity of these sensory sensitivity-related symptoms in ASD. Both of these AASP subscales are considered indicators of a “low neurological threshold” [40], indicating a tendency to overreact to sensory stimuli that are typically not bothersome. These negative correlations may appear inconsistent with the results of our group comparisons showing elevated Gln levels and Gln/Glu ratio in the ASD group. However, one plausible hypothesis is that, unlike in childhood ASD, glutamatergic neurotransmission in adults with ASD might serve a compensatory function, as discussed in the preceding paragraph. For example, while Gln and the Gln-Glu cycle are believed to regulate Glu, an increased cycle turnover could mitigate excessive glutamatergic activity and maintain a balanced excitatory tone [15, 61]. A relative decline in the efficiency of this potential compensatory mechanism, resulting in a less robust Gln-Glu cycle, may contribute to the manifestation of sensory sensitivity-related symptoms. Nevertheless, given the exploratory nature of these findings and the cross-sectional design of the current study, future research should aim to include larger sample sizes to ensure sufficient statistical power and employ longitudinal designs that encompass both childhood and adulthood. This approach will enhance the interpretation of these associations and allow for an examination of their developmental trajectories. Conversely, we did not observe any significant correlations between neurometabolite measures and SRS-2 scores. However, the absence of correlations does not exclude the potential involvement of these neurometabolites in the severity of ASD traits. Interpretation should be approached with caution, as SRS-2 scores are influenced by factors beyond ASD pathophysiology, including coping styles with social demands, self-awareness, and other elements that complicate social adjustment in individuals with ASD.

As for the regions in which significant associations with sensory symptoms were found in the ASD group, the TPJ is implicated in multisensory integration, likely playing a role in higher-level sensory perception and attention processes [62, 63]. On the other hand, the ACC is thought to process sensory information within emotional and social contexts, regulating affective reactions and forming associations between sensory stimuli and their emotional values [64]. Thus, our findings of the associations between Gln levels in the ACC and rTPJ and AASP subscale scores in ASD suggest that the atypical Gln-Glu cycle involving these regions may underlie sensory symptoms that reflect a “lower neurological threshold” in adults with ASD.

The present study has several limitations. First, while our findings remained largely unchanged after excluding participants with comorbid schizophrenia and intellectual disability, and no significant effects of comorbid ADHD or depression were observed on the neurometabolite measures examined, we cannot entirely rule out the potential influence of comorbidities on our results. Comorbid conditions such as ADHD, depression, and anxiety disorder frequently co-occur in individuals with ASD in clinical settings [65, 66], and their inclusion in the analysis would yield results more reflective of real-world conditions for these individuals. Nonetheless, it will be necessary to examine the influence of comorbid conditions of ASD on neurometabolite status in future studies with larger sample sizes. Second, 22 of the 33 individuals with ASD in our study were taking psychotropic medication. Although no significant medication effects were found in our analyses, possible impact of psychotropic medication cannot be ruled out. Third, although we successfully differentiated Gln from Glu and provided a more detailed picture of Gln-Glu cycle abnormality, which is challenging with a 3 T scanner, the MRS signal does not distinguish between intracellular and extracellular neurochemicals. Thus, caution should be exercised when interpreting the MRS analysis. Fourth, the rTPJ has functionally distinct roles across its subdivisions, including its anterior and posterior parts [45, 67]. However, the relatively large rTPJ VOI employed in our study constrains our capability to evaluate the specific functional roles of these subdivisions. Fifth, the ASD diagnosis was established through consensus among board-certified psychiatrists, including at least one psychiatrist specialized in neurodevelopmental disorders, and a clinical psychologist. However, the Autism Diagnostic Observation Schedule (ADOS) and the Autism Diagnostic Interview-Revised (ADI-R), which are considered the gold-standard diagnostic instruments for ASD, were not employed. Finally, given the cross-sectional design of this study involving adults with ASD and control subjects, neurochemical development was not investigated. Due to rapid technical advances, obtaining the MRS longitudinally over decades is not feasible. However, future research investigating the trajectories of neurometabolite status in individuals with ASD with accelerated longitudinal models would be of major interest.

In conclusion, we found significantly elevated Gln levels in the thalamus and rTPJ in adults with ASD. The Gln levels in the ACC, thalamus, and rTPJ were negatively correlated with several AASP subscale scores only in the ASD group. The findings suggest that dysregulation in the excitatory neurotransmission, potentially involving an increased cycling of the Gln-Glu circuit, may contribute to the pathophysiology of ASD.

## Supplementary information

Supplementary Information

## Data Availability

The datasets analyzed during the current study are not publicly available due to concerns regarding participant privacy and consent, but are available from the corresponding author upon reasonable request.
