# Supplementary material for: Elevated brain glutamine levels in adults with autism spectrum disorder: A 7T MRS study
Source: Mol Psychiatry. 2025 Dec 27;31(6):3062–9. doi: 10.1038/s41380-025-03440-z (PMC13190299; doi:10.1038/s41380-025-03440-z)
Supplement: Supplementary file 1 — Supplementary Information [file 41380_2025_3440_MOESM1_ESM.docx]

**Supplementary Information**

**Title:**

**Elevated brain glutamine levels in adults with autism spectrum disorder: A 7T MRS study**

**Authors:**

Manabu Kubota M.D., Ph.D.^1^, Yujiro Yoshihara M.D., Ph.D.^1^, Teruhisa Uwatoko M.D., Ph.D.^1,2^, Rin Shoji^1^, Jamie Near Ph.D.^3,4^, Masoumeh Dehghani Ph.D.^3^, Yuta Y. Aoki M.D., Ph.D.^5^, Shin-ichi Urayama^6^, Tomohisa Okada M.D., Ph.D.^6^, Toshiya Murai M.D., Ph.D.^1^

**Affiliations:**

1. Department of Psychiatry, Graduate School of Medicine, Kyoto University, Kyoto, Japan
2. University Health Center, Kyoto University of Education, Kyoto, Japan
3. Physical Sciences, Sunnybrook Research Institute, Toronto, Ontario, Canada.
4. Department of Medical Biophysics, University of Toronto, Toronto, Canada
5. Department of Psychiatry, Aoki Clinic, Tokyo, Japan
6. Human Brain Research Center, Graduate School of Medicine, Kyoto University, Kyoto, Japan

**Corresponding author:**

Manabu Kubota, M.D., Ph.D.

Department of Psychiatry, Kyoto University Graduate School of Medicine

Address: 54 Shogoin-kawahara-cho, Sakyo-ku, Kyoto 606-8507, Japan

Tel: +81-75-751-3386 Fax: +81-75-751-3246

E-mail: m_kubota@kuhp.kyoto-u.ac.jp

**Running title:** Brain glutamine levels in ASD: a 7T MRS study

**Supplementary Methods S1.** Search strategy of previous MRS studies in ASD

**Supplementary Methods S2.** Confounding effects of medication use

**Supplementary Methods S3.** Confounding effects of comorbidity

**Supplementary Results S1.** Results of literature search for previous MRS studies in ASD

**Supplementary Results S2.** Confounding effects of medication use

**Supplementary Results S3.** Confounding effects of comorbidity

**Supplementary Results S4.** Correlational analysis between neurometabolite levels and clinical measures

**Supplementary Table S1.** SNR, and CRLB of Glu, Gln, and GABA in each region in the ASD and control groups

**Supplementary Table S2.** Comparisons in mean Gln/Glu and Glu/GABA ratios between ASD and control groups

**Supplementary Figure S1.** Group comparisons of mean Gln/Glu and Glu/GABA ratios between the ASD and control groups

**Supplementary Figure S2.** Scatter plots of mean Gln/Glu ratio against AASP subscale scores for the ASD and control groups

**Supplementary References**

**Supplementary Methods S1. Search strategy for previous MRS studies in ASD**

To contextualize our current 7T MRS study involving individuals with ASD, we conducted a literature review focusing on the number of previously published MRS studies, with particular attention paid to the magnetic field strength employed. Our literature search strategy was as follows:

Initially, we reviewed English articles included in three previously published MRS meta-analyses on ASD [1-3]. Given that the literature searches for these meta-analyses were completed by September 29, 2022, October 14, 2022, and December 2010, respectively, we performed the following updated search.

Utilizing PubMed, we employed the search terms "Autism" AND ("MRS" OR "magnetic resonance spectroscopy") to identify original case-control studies on ASD published between October 15, 2022, and October 23, 2025.

**Supplementary Methods S2. Confounding effects of medication use**

To investigate the potential confounding effects of current psychotropic medication use on our findings, we performed two sets of supplementary analyses.

We firstly assessed the impact of medication status on neurometabolite measures. For those neurometabolite measures that showed significant differences between the ASD and control groups, we conducted ANCOVAs within the ASD group to evaluate the effect of medication use (yes = 1, no = 0), while controlling for age and sex.

Next, we evaluated the influence of medication on correlations between neurometabolite measures and clinical scale scores. For correlations that were significant in our original correlational analyses, we conducted partial correlational analyses, with medication status included as a covariate.

Given the exploratory nature of these post-hoc analyses, the statistical significance threshold was set at p < 0.05.

**Supplementary Methods S3. Confounding effects of comorbidity**

To investigate the potential confounding effects of comorbidity, we performed supplementary analyses, using either ADHD or depression comorbidity status (yes = 1, no = 0) as a covariate.

For neurometabolite measures that exhibited significant differences between the ASD and control groups, we performed ANCOVAs within the ASD group to evaluate the effect of comorbidity status, controlling for age and sex.

Because of the exploratory nature of these post-hoc analyses, the statistical significance threshold was set at p < 0.05.

**Supplementary Results S1. Results of the literature search for previous MRS studies in ASD**

The literature search yielded 83 MRS studies in ASD. Of these, 52 studies (63%) and 29 studies (35%) employed 3T and 1.5T MRI, respectively. Regarding studies with higher magnetic field strength, only one research group published data targeting children with ASD using 4T MRI ([4, 5]; only Glu levels were reported). Notably, in the existing literature, no published 7T MRS studies in ASD were identified.

**Supplementary Results S2. Confounding effects of medication use**

For neurometabolite measures that showed significant differences between the ASD and control groups (specifically, thalamic and rTPJ Gln levels, and the mean Gln/Glu ratio), ANCOVAs revealed no significant effects of medication use.

Partial correlational analyses confirmed that all results remained significant after controlling for medication status.

**Supplementary Results S3. Confounding effects of comorbidity**

For neurometabolite measures that demonstrated significant differences between the ASD and control groups (specifically, thalamic and rTPJ Gln levels, and the mean Gln/Glu ratio), the ANCOVAs revealed no significant effects of comorbidity.

**Supplementary Results S4. Correlational analysis between neurometabolite levels and clinical measures**

As shown in Figure 3, in the ASD group, significant negative correlations were found between the AASP Sensory Sensitivity scale and Gln levels in the ACC (Pearson’s r = -0.53, p = 0.001), thalamus (r = -0.48, p = 0.013), and rTPJ (r = -0.46, p = 0.010). Additionally, significant negative correlations were found between the AASP Sensation Avoiding scale and Gln levels in the thalamus (r = -0.48, p = 0.014) and rTPJ (r = -0.45, p = 0.014). No other significant correlations were found for either Glu, Gln, or GABA levels.

After excluding one individual with comorbid schizophrenia from the ASD group and another with comorbid mild intellectual disability, these results remained significant; with the exception that the correlations between the thalamic Gln levels and the AASP Sensory Sensitivity (r = -0.48, p = 0.018) and Sensation Avoiding (r = -0.47, p = 0.019) scales became trend levels while r values remained largely similar, likely due to the decreased sample size.

To further validate the results of the correlations between Gln levels and symptom scale scores, we implemented the false discovery rate (FDR) correction across 15 comparisons (3 VOIs × 5 symptom scores, including the SRS-2 total score and the four subscales of AASP). The analysis revealed that all five negative correlations in the ASD group, initially reported as significant, persisted after correction, while no additional correlations achieved significance.

**Supplementary Table S1. SNR, and CRLB of Glu, Gln, and GABA in each region in the ASD and control groups**

|  | ASD (N = 33) | | |  | Controls (N = 52) | | |  | Statistics | |
| --- | --- | --- | --- | --- | --- | --- | --- | --- | --- | --- |
|  | n | Mean | S.D. |  | n | Mean | S.D. |  | t | p |
| SNR |  |  |  |  |  |  |  |  |  |  |
| ACC | 33 | 59.0 | 10.1 |  | 51 | 59.2 | 9.4 |  | 0.10 | 0.92 |
| Thalamus | 29 | 42.1 | 13.2 |  | 45 | 44.0 | 10.5 |  | 1.21 | 0.23 |
| rTPJ | 30 | 65.5 | 15.6 |  | 50 | 66.3 | 13.6 |  | 0.23 | 0.82 |
|  |  |  |  |  |  |  |  |  |  |  |
| CRLB: Glu |  |  |  |  |  |  |  |  |  |  |
| ACC | 33 | 2.06 | 0.35 |  | 51 | 2.06 | 0.31 |  | -0.02 | 0.98 |
| Thalamus | 29 | 2.59 | 1.02 |  | 45 | 2.42 | 0.78 |  | -0.78 | 0.44 |
| rTPJ | 30 | 2.20 | 0.48 |  | 50 | 2.14 | 0.45 |  | -0.56 | 0.58 |
|  |  |  |  |  |  |  |  |  |  |  |
| CRLB: Gln |  |  |  |  |  |  |  |  |  |  |
| ACC | 33 | 6.24 | 2.56 |  | 50 | 6.20 | 1.36 |  | -0.10 | 0.92 |
| Thalamus | 26 | 8.54 | 2.28 |  | 44 | 9.50 | 2.82 |  | 1.47 | 0.15 |
| rTPJ | 30 | 7.73 | 3.22 |  | 49 | 8.06 | 2.63 |  | 0.49 | 0.62 |
|  |  |  |  |  |  |  |  |  |  |  |
| CRLB: GABA |  |  |  |  |  |  |  |  |  |  |
| ACC | 31 | 10.71 | 1.94 |  | 47 | 10.85 | 2.55 |  | 0.26 | 0.79 |
| Thalamus | 24 | 12.17 | 3.43 |  | 38 | 12.74 | 3.45 |  | 0.63 | 0.53 |
| rTPJ | 26 | 9.23 | 2.21 |  | 45 | 10.31 | 3.48 |  | 1.60 | 0.12 |

Abbreviations: ACC, anterior cingulate cortex; ASD, autism spectrum disorder; CRLB, Cramér–Rao lower bound; SNR, signal-to-noise ratio; GABA, γ-aminobutyric acid; Glu, glutamate; Gln, glutamine; rTPJ, right temporo-parietal junction

**Supplementary Table S2. Comparisons in mean Gln/Glu and Glu/GABA ratios between ASD and control groups**

|  | ASD (N = 33) | | |  | Controls (N = 52) | | |  | Effect of diagnosis | |
| --- | --- | --- | --- | --- | --- | --- | --- | --- | --- | --- |
|  | n | Mean | S.D. |  | n | Mean | S.D. |  | F | p |
|  |  |  |  |  |  |  |  |  |  |  |
| Mean Gln/Glu | 24 | 0.25 | 0.05 |  | 42 | 0.23 | 0.04 |  | 6.14 | 0.016^1)^ |
| Mean Glu/GABA | 20 | 6.19 | 0.60 |  | 32 | 6.35 | 0.70 |  | 0.31 | 0.58 |

1) p < 0.05

Mean Gln/Glu and Glu/GABA ratios were calculated across the three regions (anterior cingulate cortex, thalamus, and right temporo-parietal junction).

Abbreviations: ASD, autism spectrum disorder; Glu, glutamate; Gln, glutamine; GABA, γ-aminobutyric acid; S.D., standard deviation

**Supplementary Figure S1. Group comparisons of mean Gln/Glu and Glu/GABA ratios between the ASD and control groups**

**
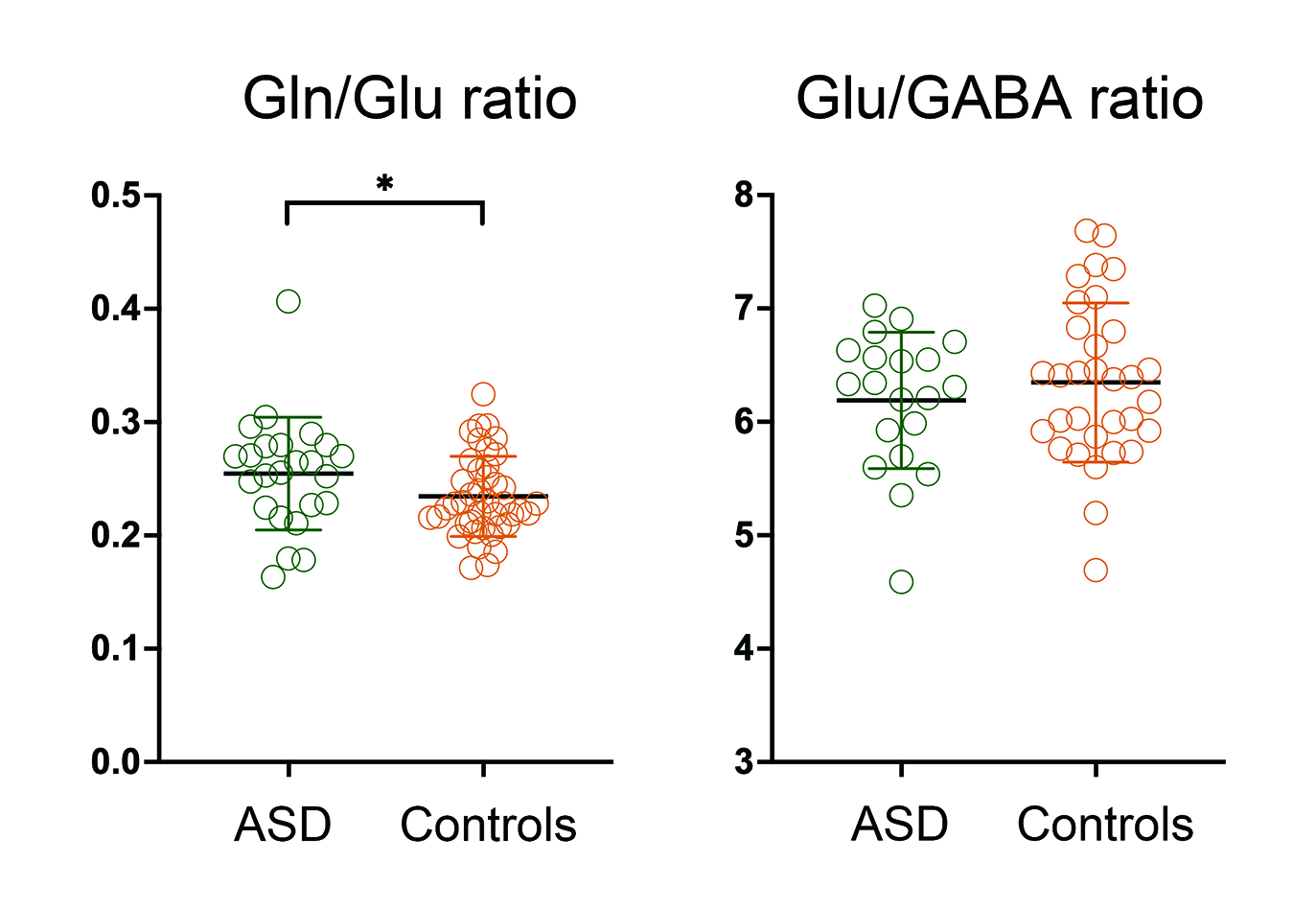
**

Error bars represent standard deviation.

*p < 0.05

Mean Gln/Glu and Glu/GABA ratios were calculated across the three regions (anterior cingulate cortex, thalamus, and right temporo-parietal junction)

Abbreviations: ASD, autism spectrum disorder; Glu, glutamate; Gln, glutamine; GABA, γ-aminobutyric acid

**Supplementary Figure S2. Scatter plots of mean Gln/Glu ratio against AASP subscale scores for the ASD and control groups**

**
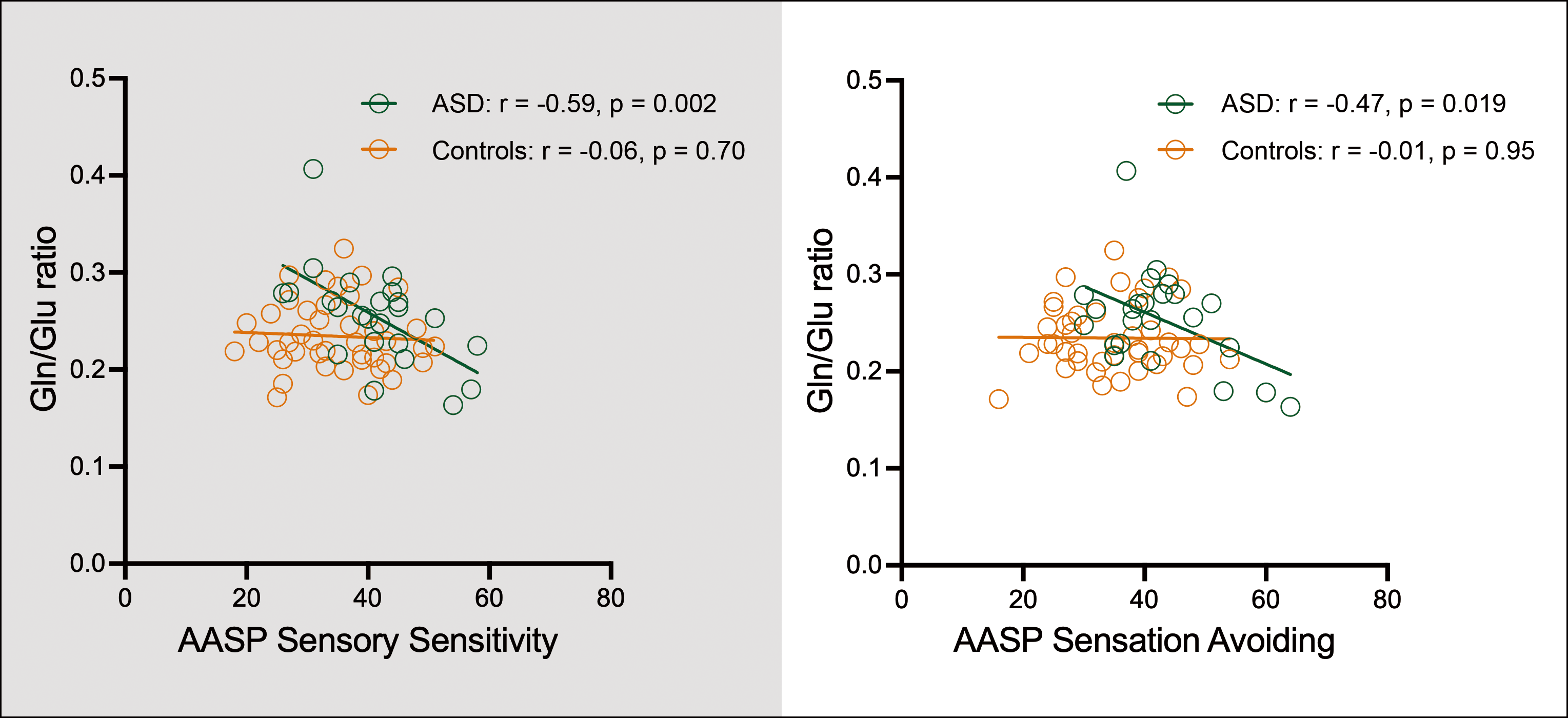
**

Mean Gln/Glu ratio was calculated across the three regions (anterior cingulate cortex, thalamus, and right temporo-parietal junction).

The gray background indicates a significant group difference in the relationship between the Gln/Glu ratio and the AASP Sensory Sensitivity scores, based on Fisher's z-transformation of the correlation coefficient (z = 2.3, p = 0.02).

Abbreviations: AASP, Adolescent/Adult Sensory Profile; ASD, autism spectrum disorder; Glu, glutamate; Gln, glutamine

**Supplementary References**

1. Thomson AR, Pasanta D, Arichi T, Puts NA. Neurometabolite differences in autism as assessed with magnetic resonance spectroscopy: A systematic review and meta-analysis. *Neurosci Biobehav Rev* 2024; **162:** 105728.

2. Du Y, Chen L, Yan MC, Wang YL, Zhong XL, Xv CX *et al.* Neurometabolite levels in the brains of patients with autism spectrum disorders: A meta-analysis of proton magnetic resonance spectroscopy studies (n = 1501). *Mol Psychiatry* 2023; **28**(7)**:** 3092-3103.

3. Aoki Y, Kasai K, Yamasue H. Age-related change in brain metabolite abnormalities in autism: A meta-analysis of proton magnetic resonance spectroscopy studies. *Transl Psychiatry* 2012; **2:** e69.

4. Joshi G, Biederman J, Wozniak J, Goldin RL, Crowley D, Furtak S *et al.* Magnetic resonance spectroscopy study of the glutamatergic system in adolescent males with high-functioning autistic disorder: A pilot study at 4t. *Eur Arch Psychiatry Clin Neurosci* 2013; **263**(5)**:** 379-384.

5. Joshi G, Gonenc A, DiSalvo M, Faraone SV, Ceranoglu TA, Yule AM *et al.* Memantine to treat social impairment in youths with autism spectrum disorder: A randomized clinical trial. *JAMA Netw Open* 2025; **8**(10)**:** e2534927.
